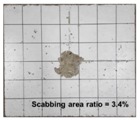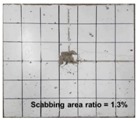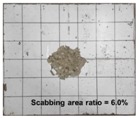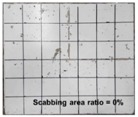# Strain Behavior of Concrete Panels Subjected to Different Nose Shapes of Projectile Impact

**DOI:** 10.3390/ma11030409

**Published:** 2018-03-09

**Authors:** Sangkyu Lee, Gyuyong Kim, Hongseop Kim, Minjae Son, Gyeongcheol Choe, Jeongsoo Nam

**Affiliations:** Department of Architectural Engineering, Chungnam National University, 99 Daehak-ro, Yuseong-gu, Daejeon 34134, Korea; lsg2357@naver.com (S.L.); oliver-kahn12@nate.com (H.K.); minjae931226@naver.com (M.S.); speed1382@nate.com (G.C.); j.nam@cnu.ac.kr (J.N.)

**Keywords:** projectile nose shape, strain behavior, impact, fiber-reinforced concrete

## Abstract

This study evaluates the fracture properties and rear-face strain distribution of nonreinforced and hooked steel fiber-reinforced concrete panels penetrated by projectiles of three different nose shapes: sharp, hemispherical, and flat. The sharp projectile nose resulted in a deeper penetration because of the concentration of the impact force. Conversely, the flat projectile nose resulted in shallower penetrations. The penetration based on different projectile nose shapes is directly related to the impact force transmitted to the rear face. Scabbing can be more accurately predicted by the tensile strain on the rear face of concrete due to the projectile nose shape. The tensile strain on the rear face of the concrete was reduced by the hooked steel fiber reinforcement because the hooked steel fiber absorbed some of the impact stress transmitted to the rear face of the concrete. Consequently, the strain behavior on the rear face of concrete according to the projectile nose shape was confirmed.

## 1. Introduction

**Figure 1:**
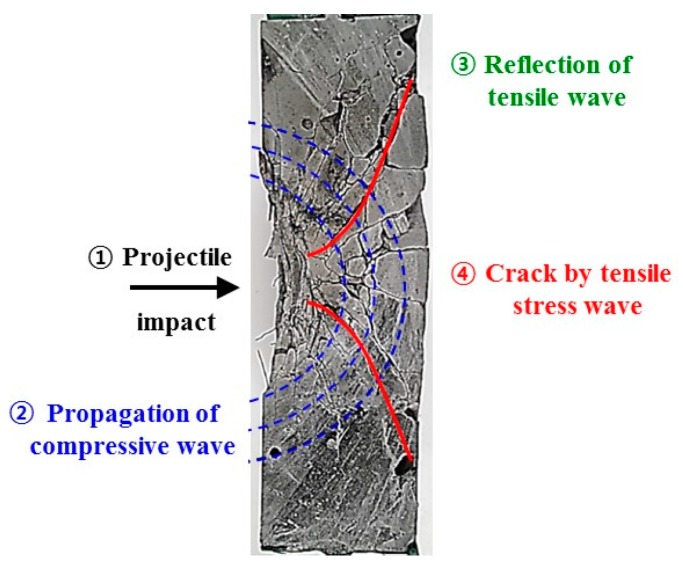
Damage regions and propagation of the elastic stress wave in the concrete subjected to impact forces.

Front-side failure is obtained when a projectile strikes concrete. At this point, a compressive stress wave is generated and radially transmitted to the rear face of the concrete. This compressive stress wave is reflected as a tensile stress wave when it reaches the rear face. This action causes a decrease in the magnitude of the compressive wave and an increase in the magnitude of the tensile wave. Cracks and fractures will occur if the generated tensile stress exceeds the dynamic tensile strength of the concrete and the strain limits at any point in the concrete matrix (Figure 1) [1]. Prior research classified the local fracture modes of concrete as penetration, scabbing, and perforation failure [1,2,3,4].

The suppression of a missile perforation failure is generally considered a priority in the protective design of military facilities. In civilian applications, maximizing human safety and minimizing property damage in the event of concrete scabbing are a priority for general infrastructure design. Therefore, a concrete specimen thickness that can prevent scabbing and perforation by projectile impact is considered fundamental in protection design.

**Figure 2:**
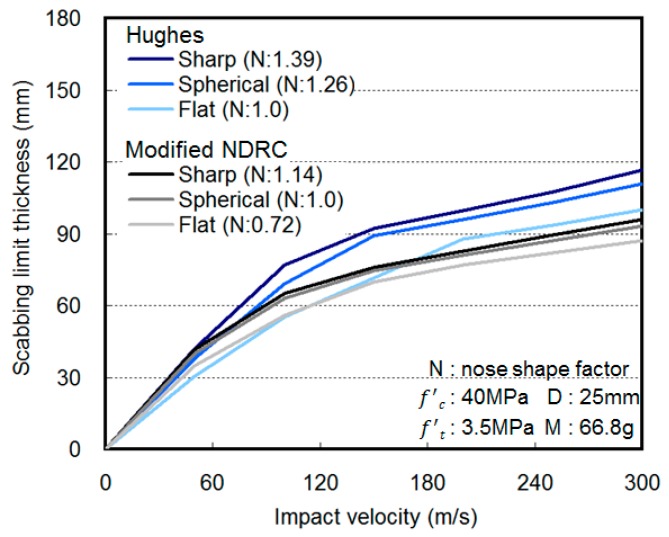
Scabbing limit thickness by projectile nose shape.

Various empirical formulae have been proposed by researchers to predict the penetration depth, scabbing limit thickness, and perforation limit thickness of concrete based on various impact conditions. Table 1 presents the modified National Defense Research Committee (NDRC), Hughes, Haldar and Hamieh, and United Kingdom Atomic Energy Authority (UKAEA) prediction formulae for concrete penetration depth and scabbing limit thickness. The local fracture of the concrete caused by a projectile impact is primarily affected by two factors: (1) impact conditions (i.e., projectile diameter and mass, projectile nose shape, and impact velocity) and (2) material properties of concrete (i.e., compressive strength, flexural tensile strength, and specimen thickness). The existing empirical formulae focus on these two factors in predicting the penetration depth and the limit thickness, as shown in Figure 2 [5,6,7,8,9,10].

The application ranges of these empirical formulae are limited by the data ranges associated with their derivation. Regression techniques were applied to the development of equations for calculating the scabbing and perforation limit thicknesses based on the penetration depth [11,12]. The accuracy of these empirical formula was recently improved, and research was conducted to widen their application range.

Expanded applications include considering different projectile nose shapes (e.g., flat, blunt, spherical, sharp, etc.) in predicting local fracture. Several researchers applied nose shape considerations to the penetration depth calculations. Almusallam et al. [13] used a biconical projectile to determine the impact resistance of a fiber-reinforced concrete (RC) composite slab based on the blending ratio of steel and plastic fiber. The highest front- and rear-face failure areas and crack reduction effects were observed when the steel-to-plastic fiber ratio was 2:1. Tai [14] conducted an impact test using a flat projectile to evaluate the flexural tensile performance and impact resistance after steel fiber reinforcement in normal concrete (NC) and reactive-powder concrete (RPC). The RPC showed a much greater improvement in flexural tensile performance and impact resistance compared with the NC following steel fiber reinforcement. Siddiqui et al. [15] performed impact tests using ogive and biconical projectiles striking a steel-plate-reinforced RC slab. The perforation limit velocity for the ogive projectile was lower than that of the biconical projectile.

Focusing more on formulation, Wen et al. [16] proposed a penetration depth equation (i.e., the University of Manchester Institute of Science and Technology (UMIST) formula) that included quasi-static and dynamic resistance parameters and was appropriate for various projectile nose shapes and a wide velocity range. In a subsequent study, Wen et al. [17] compared the UMIST formula, which was derived using a flat projectile, with external experimental data to develop an advanced UMIST formula that yielded reduced margins of error in the UMIST formula through variable segmentation. Shiu et al. [12] performed an impact analysis using conical and flat projectiles and discrete element methods (DEM). The predicted results obtained were consistent with the experimental results, thereby confirming the applicability of DEM to penetration depth prediction based on the projectile nose shape.

Thus, the empirical formulae for the concrete subjected to projectile impact predicts the penetration depth according to the projectile nose shape, and the scabbing depth is predicted by the predicted value of the penetration depth. Prior studies mainly investigated the improvement of the prediction accuracy concerning normal concrete without fiber reinforcement according to the projectile nose shape and analyzed the fracture properties of concrete, which were not present in the empirical formula, using the projectile nose shape.

However, the behavior of the fracture on the rear face of the concrete caused by the projectile impact is affected by the stress wave transmitted to the rear face of the concrete. Therefore, the generation and transfer distribution of the impact stress wave differ depending on the projectile nose shape and considerably influence the fracture on the rear face of the concrete.

In addition, fiber-reinforced concrete restrains the fracture on the rear face of the concrete by the offset of the impact stress wave and the reduction in the tensile strain caused by the improvement of the flexural tensile performance and toughness (energy-absorbing capacity) [18,19].

In this study, the fracture pattern and the crack distribution on the cross section of the concrete were observed after impact by three types of projectile (i.e., sharp, hemispherical, and flat nose shape) on nonreinforced and hooked steel fiber-reinforced concrete. The fracture depth and the crater diameter were evaluated. Furthermore, an analysis of the strain behavior on the rear face of the concrete and the distribution of the impact stress according to the projectile nose shape was performed.

## 2. Experimental Program

**Figure 3:**
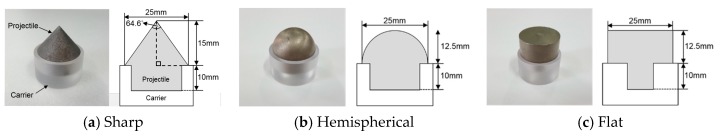
Configuration of projectile nose shapes.

Table 2 shows the experimental design, and Figure 3 shows the configuration of projectile nose shapes used in this study. The sharp projectile from the carrier had a diameter of 25 mm, a nose length of 15 mm, and a nose angle of 64.6°. In addition, the hemispherical and flat projectiles had a diameter of 25 mm. The mass of each projectile including the carrier was 66.8 g. The projectiles were made of SKD11 (tensile strength: 820–850 σb/MPa, yield strength: 815–875 σb/MPa). The impact velocity was controlled at 170 m/s, and the carrier was detached right before impact. The fabricated concrete panel had a 700 mm width, 600 mm length, and 50 and 60 mm thicknesses. Rebar was not used in the concrete panel.

**Figure 4:**
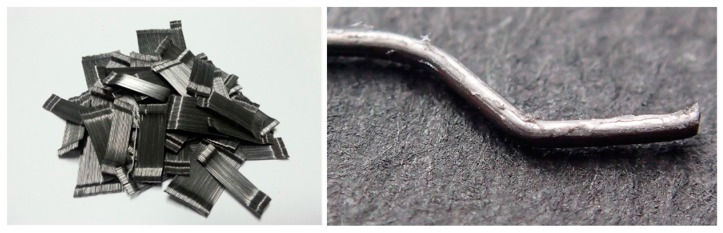
Shape of the hooked steel fiber.

Table 3 gives the mix proportions of concrete. Nonreinforced concrete (NC) and hooked steel fiber-reinforced concrete (HSFRC) panels, which were subjected only to hemispherical projectile impact in a previous study, were fabricated [20,21]. The fiber volume fraction was 1.0%. A 0.40 water/binder (W/B) ratio was used to achieve a compressive strength of 50 MPa (design compressive strength) at the age of 28 days. Table 4 presents the mechanical properties of the materials. Ordinary Portland cement and fly ash were used in the study. River sand with a density of 2.61 g/cm^3^ and a water absorption of 0.81% was used as the fine aggregate. Crushed gravel with a density of 2.65 g/cm^3^, a water absorption of 0.76%, and a maximum size of 20 mm was used as the coarse aggregate. A polycarboxylic acid-based superplasticizer was used to achieve sufficient concrete slump. The hooked steel fiber had a length of 30 mm, a diameter of 0.5 mm, an aspect ratio of 60, and a tensile strength of 1140 MPa. Figure 4 illustrates the shape of the hooked steel fiber. Table 5 summarizes the details regarding the compressive, flexural, and split tensile strengths of the concrete. In addition, each value is an average value for three tests. For the compressive strength, that of HSFRC was higher than that of NC. This seems to have been due to the constraining effect of the reinforced fibers.

A twin-shaft mixer was used for concrete mixing. After dry mixing for 30 s with aggregate and binder, the process was finished by dry mixing for 90 s with the hooked steel fibers. Final mixing was then carried out for 300 s with admixture and water. Concrete compaction was performed by a vibrator. A slump value of approximately 150 mm was secured in all fresh concrete. Segregation was not observed.

**Figure 5:**
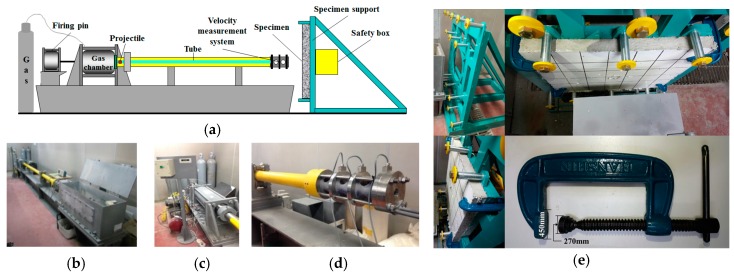
High-velocity impact test device. (**a**) Overall composition of high-velocity impact test device **(b)** Machine exterior; (**c**) Launch section; (**d**) Velocity measurement system; (**e**) Support and clamp.

Figure 5 shows the high-velocity projectile impact test device (gas pressure type, R&D Support Co., Ltd., Tokyo, Japan) used in this study. This device used a temporary spray method. Figure 5a,b show the overall composition of the device and machine exterior. The gas chamber shown in Figure 5c was filled with nitrogen gas at a pressure of 1.5 MPa. The specimen was fixed using clamps on the specimen support, and the projectile was launched with a velocity of 170 m/s. The impact velocity was measured using the velocity measurement system shown in Figure 5d. The residual velocity was not measured because the experiments were planned for “no perforation”. Figure 5e illustrates the support and clamp.

**Figure 6:**
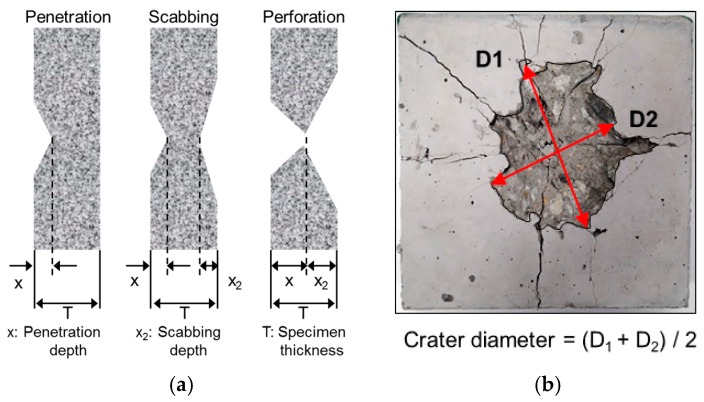
Measurement of fracture depth and crater diameter. (**a**) Failure mode and fracture depth; (**b**) Crater diameter.

Figure 6 depicts the measurement methods used to determine the failure mode, fracture depth, and crater diameter. The crater diameter was determined as the average of the maximum and minimum diameters (*D*_1_ and *D*_2_, respectively).

**Figure 7:**
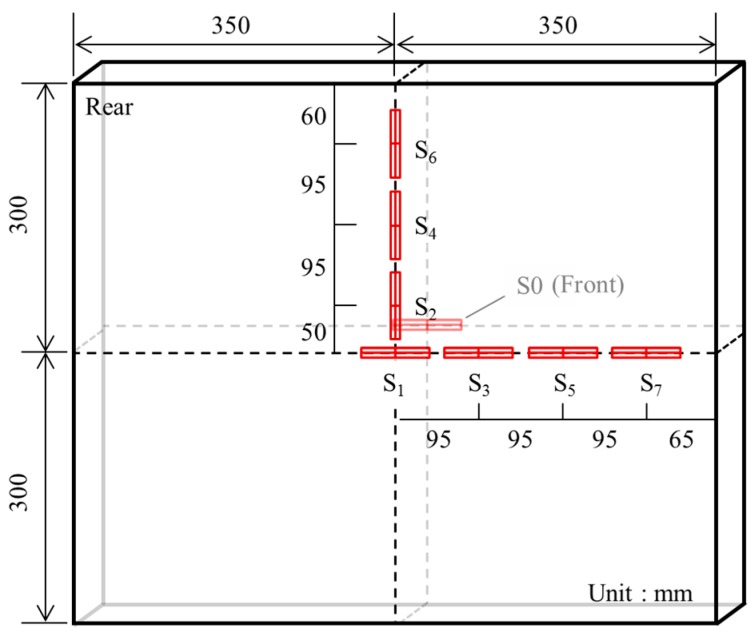
Position of strain gauges. (*S*_0_: Center of front face, *S*_1_~*S*_7_: Rear face, Sampling rate: 200,000 Hz).

Figure 7 presents the position of the strain gauge (PL-60, Tokyo Sokki Kenkyujo Co., Ltd., Tokyo, Japan) used to measure the rear-face strain. One gauge was attached at the projectile impact point (*S*_0_) on the front face of the concrete panel to check the initial impact time. Seven additional gauges (*S*_1_–*S*_7_) were attached to the rear face of the concrete panel at distances of 0–285 mm from the center of the rear face. The sampling rate for the strain measurements was set to 200,000 Hz.

## 3. Results and Discussion

### 3.1. Appearance of Fracture on Concrete

**Figure 8:**
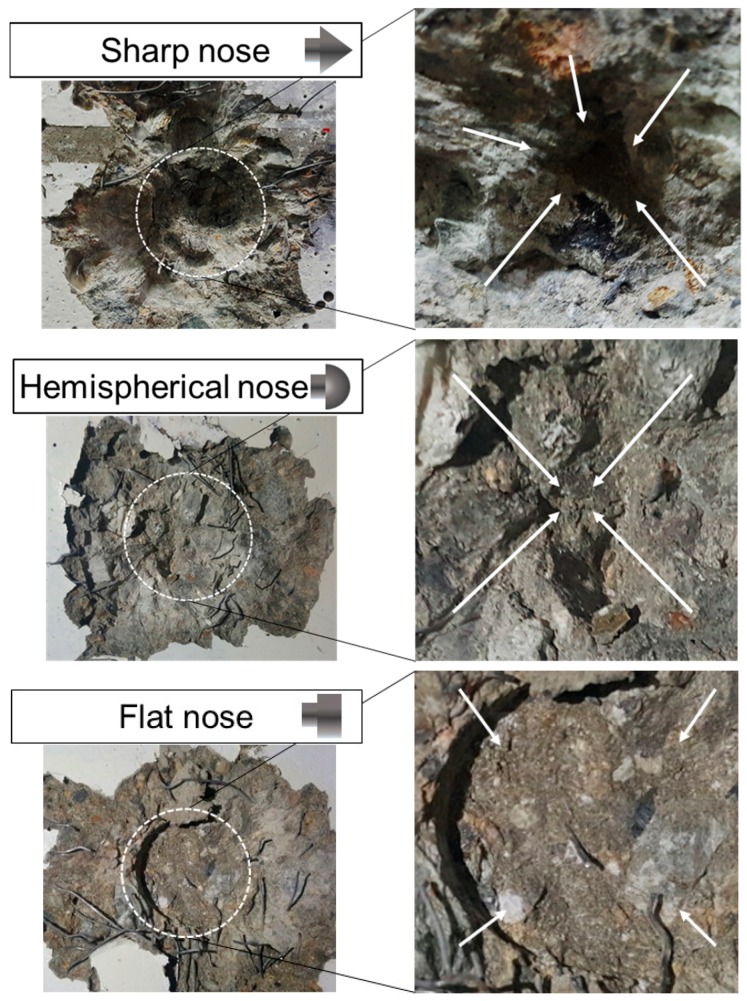
Impact fracture on the front face.

Figure 8 shows the impact fracture on the front face of the concrete panels by each projectile nose shape.

The sharp nose shape caused the deepest penetration because of its sharp tip and small surface area. Conversely, the flat nose shape caused the shallowest penetration. The penetration depth for the hemispherical nose shape was between the sharp and flat penetration depths. Each projectile nose shape produced a similarly shaped indentation in the concrete.

Table 6 lists the impact fracture on the rear face of the concrete panels following the projectile impact. For the 50 mm thick specimens, scabbing occurred in all the NC and HSFRC panels impacted by the sharp, hemispherical, and flat projectiles. Although both concrete types showed scabbing, the fiber-reinforced concrete had a smaller affected area. The fragment delamination was suppressed by the fiber–matrix interaction [22,23,24,25,26].

For the 60 mm thick specimens, scabbing occurred in the NC panels impacted by the hemispherical and flat projectile impacts. Scabbing did not occur following the sharp projectile impact. The impact by the hemispherical nose shape resulted in a scabbing area larger than that caused by the flat nose shape. Scabbing did not occur in the HSFRC panels because of the higher flexural tensile strength [27,28,29].

**Figure 9:**
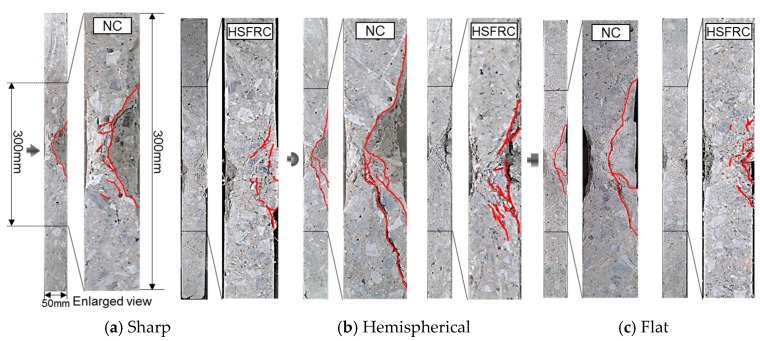
Cross section of the concrete panel (thickness 50 mm).

Figure 9 shows the cross section of the 50 mm thick concrete panel. For the sharp nose projectile in the NC, cracks with a small slope were developed, and scabbing was small because the impact stress was concentrated on the center of the cross section of the concrete panel. Meanwhile, scabbing for the flat nose projectile was small because of the impact stress dispersion. In contrast, for the hemispherical nose projectile, the region where scabbing occurred was the largest because of the large slope crack.

The cracks inside the HSFRC, which were caused by the projectile impact, were reduced because the flexural tensile performance was improved by the reinforcing fibers. In the case of the hemispherical nose projectile, scabbing was restrained because the large slope crack could not reach the backside.

### 3.2. Fracture Depth and Crater Diameter

**Figure 10:**
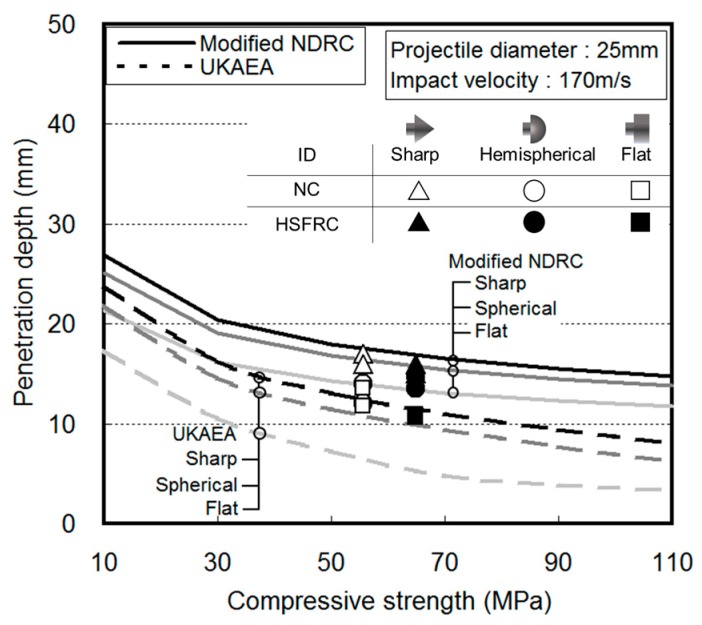
The experimentally observed and predicted penetration depths.

The measured penetration depths for the various projectile nose shapes were compared with the values predicted using the modified NDRC and UKAEA formulae (Figure 10). As presented in Table 1, the modified NDRC and UKAEA formulae predicted the local fracture based on a projectile nose shape factor. According to these relationships, the penetration depth increased as the projectile nose shape factor increased. An impact by a sharp projectile, which had the highest nose shape factor, caused the deepest penetration. The impact force was concentrated during collision and the penetration depth increased because the impact area of a sharp projectile was small owing to the sharp nose shape. The penetration depth tended to decrease as the nose shape factor decreased. The impact by the flat projectile caused a shallower penetration because the large impact area distributed the impact force more. The penetration depths measured in this study were similar to those predicted by the modified NDRC formula. The penetration trend according to the projectile nose shape was similar to the results predicted by both NC and HSFRC. However, the penetration depth values were slightly shallower in HSFRC.

**Figure 11:**
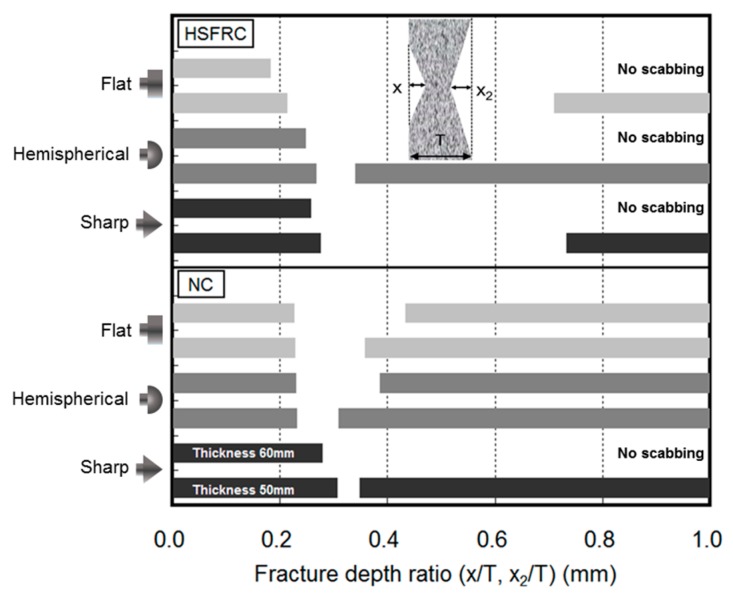
Fracture depth ratio.

Figure 11 illustrates the front- and rear-face fracture depths as a proportion of the specimen thickness for the various projectile nose shapes. The penetration ratio increased as the projectile nose shape factor increased. Although the sharp nose shape produced a higher penetration ratio than did either the hemispherical or flat projectile nose shape, it did not result in significant scabbing. The hemispherical nose shape, which caused a penetration shallower than that of the sharp nose shape, resulted in the deepest scabbing.

**Figure 12:**
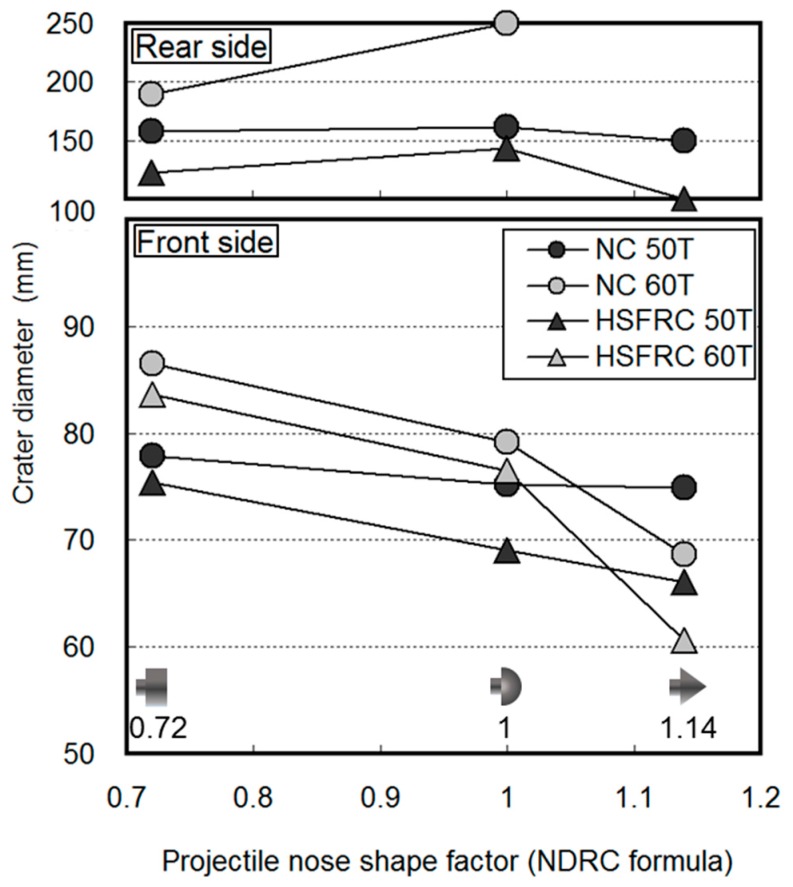
Crater diameter.

Figure 12 shows the front- and rear-face crater diameters. The values from the modified NDRC formula were used as the projectile nose shape factors. The largest front-face crater diameter was produced by the flat nose shape projectile with the smallest nose shape factor and increased as the concrete thickness increased. Smaller projectile nose shape factors were thought to cause larger front-face crater diameters because of the large shock area to the front face.

The hemispherical projectile nose shape generated the largest rear-face crater diameter. Given its large impact area and shallower penetration, the rear-face crater diameter generated by the flat nose shape was thought to be smaller because the impact force transmitted to the rear face was distributed over a larger area. Conversely, the rear-face crater diameter generated by the sharp nose shape was thought to be the smallest because the impact force was concentrated in a smaller area, given its small impact area and deep penetration.

Irrespective of the projectile nose shape, the front- and rear-face crater diameters were smaller for the HSFRC panels than for the NC panels. This result was attributed to the higher flexural tensile strength and the lower potential for scabbing in the fiber-reinforced concrete.

### 3.3. Rear-Face Strain Behavior

**Figure 13:**
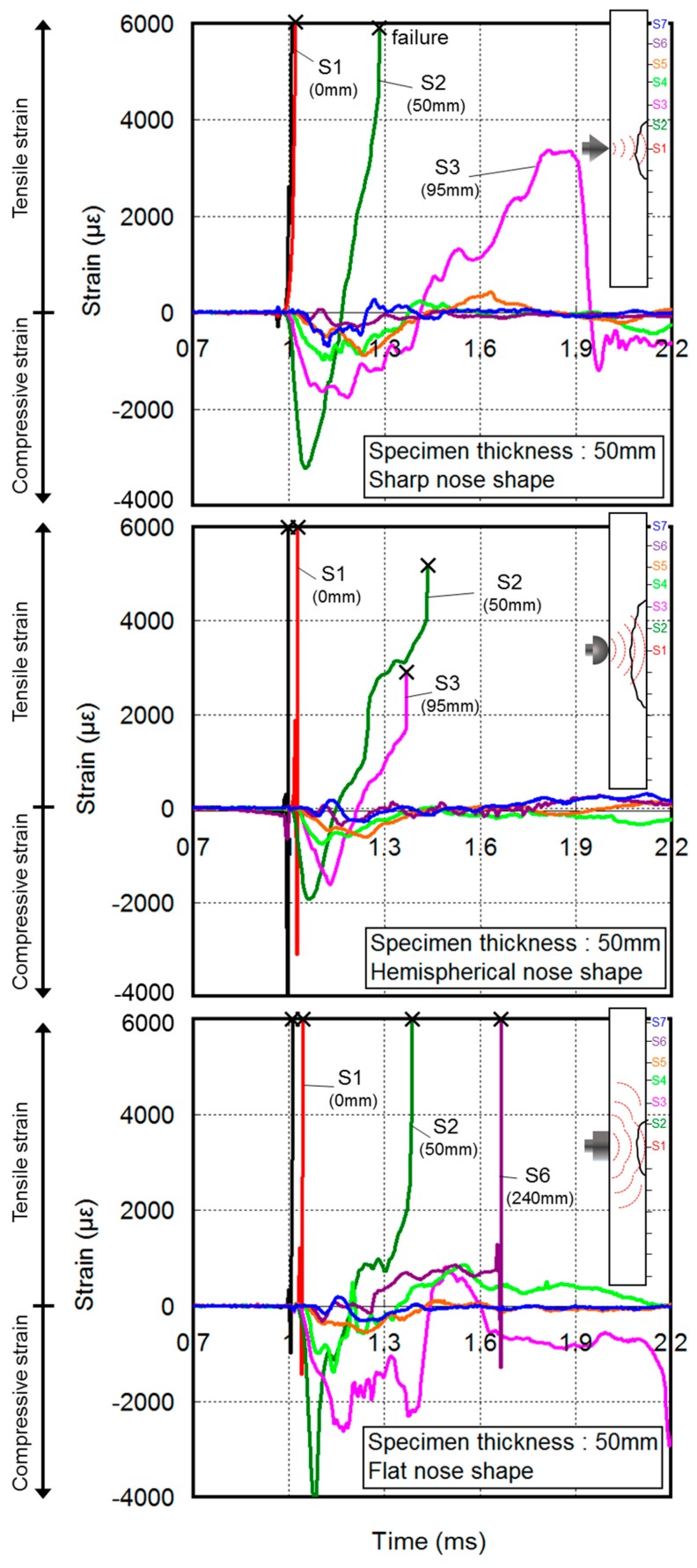
Rear-face strain curve of NC.

Figure 13 presents the rear-face strain for the 50 mm thick concrete specimens because of the projectile impact. The gauge on the front face was broken by the projectile impact. Thus, the impact start time was set to 1 ms. In addition, for the fracture behavior on the rear face of the concrete subjected to the projectile impact, a compressive stress wave was transmitted to the rear face when the concrete was impacted by a projectile, where it was transformed into a tensile stress wave. The rear-face compressive strain at the initial stage of impact was converted into tensile strain over time. The gauges on the rear face were broken as the concrete began to crack and fracture.

**Figure 14:**
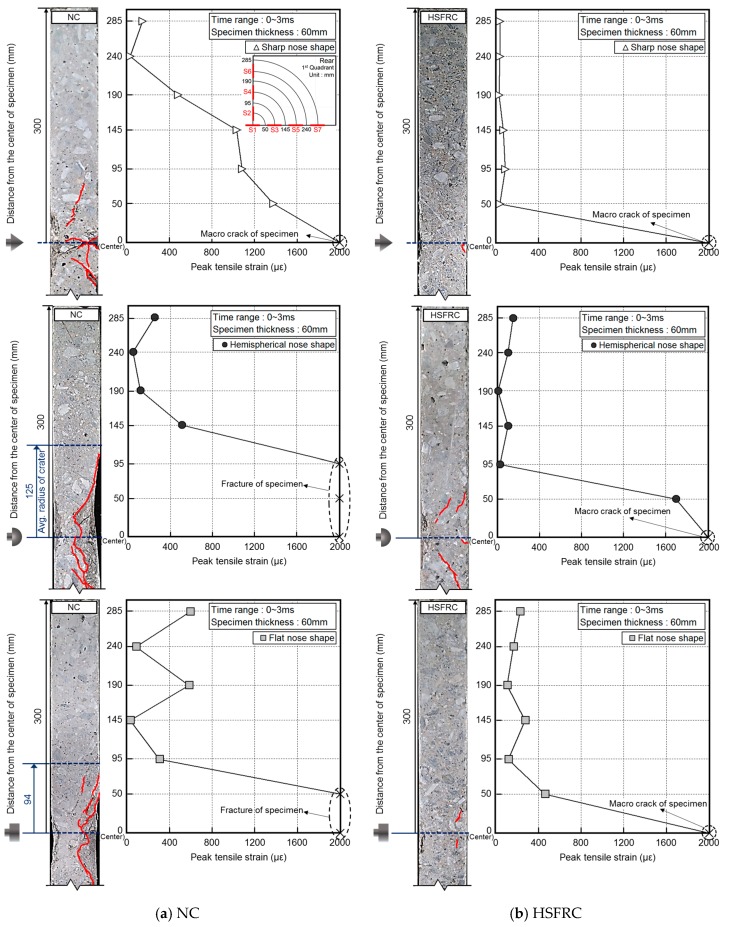
Relationship between cracks on the cross section and peak tensile strain on the rear face.

Figure 14 shows the relationship between the cracks on the cross section of the concrete and the peak tensile strain on the rear face from the time–strain history curve for the 60 mm thick concrete at the various gauge positions. Following the sharp projectile impact, the *S*_1_ gauge (0 mm from the specimen center) was broken by concrete cracking. Cracking occurred inside the specimen near the *S*_2_–*S*_3_ gauges (50–95 mm from the specimen center), and the tensile strain was minimal near the *S*_5_–*S*_7_ gauges (190–285 mm from the specimen center). Similar to that of NC, the tensile strain of the *S*_1_ gauge for the HSFRC was high because of the occurrence of cracks from the concentrated impact stress at the center on the rear face of the concrete. However, the tensile strain sharply decreased after the *S*_1_ gauge.

Following the hemispherical projectile impact, the *S*_1_–*S*_3_ gauges (0–95 mm from the specimen center) were broken by the large tensile strain, and the scabbing area was the largest. Compared to that of NC, the tensile strain for the HSFRC was observed in a narrow area because the impact stress affecting the center on the rear face of the concrete was reduced by the fiber reinforcement.

The tensile strain caused by the flat projectile impact at *S*_3_ and *S*_4_ (95 and 145 mm from the specimen center, respectively) was lower than the strains owing to the sharp and hemispherical projectile impacts, but higher for *S*_5_–*S*_7_ (190–285 mm from the specimen center). The peak tensile strain caused by the flat projectile impact in *S*_5_–*S*_7_ was relatively higher than that of the sharp and hemispherical projectile impacts because the impact stress was dispersed in a wide range owing to the large impact area of the flat projectile. For the HSFRC, a tensile strain relatively higher than those of the sharp and hemispherical nose projectiles occurred at the *S*_3_ gauge.

The impact force for the sharp nose shape was concentrated at the middle of the rear face, but scabbing did not occur because the concentrated area of the impact stress was lower in the 60 mm thick specimens compared to that in the 50 mm thick specimens. The impact force for the hemispherical nose shape was also concentrated at the middle, but over a larger area than in the sharp impact case. Meanwhile, the impact stress for the flat nose shape was distributed to the edges of the specimens.

The strain behavior and the stress wave transmitted to the rear face varied based on the projectile nose shape and were thought to have a significant effect on the crack distribution of the cross section of the concrete panel and the crater diameter on the rear face of the concrete. Irrespective of the projectile nose shape, the overall tensile strain magnitude was lower for the HSFRC than for the NC panel. The reinforcing fibers were thought to absorb the impact force, thereby reducing the overall tensile strain.

## 4. Conclusions

This study evaluated the influence of projectile nose shape on the strain behavior and fracture properties of fiber-reinforced concrete panels due to projectile impact. The following conclusions can be drawn:

- The sharp projectile nose resulted in a deeper penetration than hemispherical and flat projectile nose by up to about 36% because of the concentration of the impact force. Conversely, the flat projectile nose resulted in shallower penetrations because of the distribution of the impact force. In addition, the scabbing depth and crater diameter on the rear face of the concrete were larger for hemispherical projectile impact than for other projectiles. Therefore, if the scabbing occurs in concrete due to projectile impact, it is expected that the amount of the fragment by hemispherical projectiles will be greatest.
- The tensile strain on the rear face obtained from the experimental result was widely distributed as the projectile nose shape became blunter. The strain history on the rear face also differed in each projectile impact because the propagation path of the impact stress that transmitted to the rear face of the concrete was changed by the projectile nose shape. Therefore, the projectile nose shape had a significant effect on the crack distribution and the crater diameter on the rear face of the concrete.
- The penetration based on different projectile nose shapes was directly related to the impact force transmitted to the rear face. Considering the additional effect of the tensile strain on the rear face in predicting the fracture behavior, the scabbing caused by the projectile nose shape can more accurately be predicted. Furthermore, the crater diameter on the rear face of the concrete can be predicted.
- The tensile strain on the rear face of the concrete was reduced by the reinforcement of the hooked steel fiber because of the absorption of the impact stress transmitted to the rear face of the concrete by the hooked steel fiber. Furthermore, the strain history on the rear face is thought to more effectively reflect the deformability of the fiber-reinforced concrete owing to the impact load.

## Figures and Tables

**Table 1:** Empirical formulae.

Modified National Defense Research Committee NDRC [5]	
Penetration depth	Scabbing limit thickness
$G=3.813\times 10^{-5}\frac{NM}{D\sqrt{{f^{\prime }}_{c}}}{\left(\frac{V_{0}}{D}\right)}^{1.8}$ $G={\left(\frac{x}{2d}\right)}^{2}\;\left(\frac{x}{d}\leq 2\right),\;G=\frac{x}{d}-1\;\left(\frac{x}{d}>2\right)$	$\frac{s}{d}=7.91\left(\frac{x}{d}\right)-5.06{\left(\frac{x}{d}\right)}^{2}\;\left(\frac{x}{d}\leq 0.65\right)$ $\frac{s}{d}=2.12+1.36\left(\frac{x}{d}\right)\;\left(0.65<\frac{x}{d}\leq 11.75\right)$
Hughes [6]	
Penetration depth	Scabbing limit thickness
$\frac{s}{d}=0.19\frac{N_{h}I}{S},\;I=\frac{M{V_{0}}^{2}}{d^{3}f_{t}}$ S=1.0+12.3 ln(1.0+0.03I)	$\frac{s}{d}=5.0\left(\frac{x}{d}\right)\;\left(\frac{x}{d}<0.7\right)$ $\frac{s}{d}=1.74\frac{x}{d}+2.3\;\left(\frac{x}{d}\geq 0.7\right)$
Haldar and Hamieh [7]	
Penetration depth	Scabbing limit thickness
$I=\left(\frac{MNV^{2}}{d^{3}f_{c}}\right)$ $\frac{x}{d}=-0.0308+0.2251I\;\left(0.3\leq I\leq 4.0\right)$ $\frac{x}{d}=0.6740+0.0567I\;\left(4.0<I\leq 21.0\right)$ $\frac{x}{d}=1.1875+0.0299I\;\left(21.0<I\leq 455\right)$	$\frac{s}{d}=3.3437+0.342I\;\left(21\leq \frac{x}{d}\leq 385\right)$
United Kingdom Atomic Energy Authority (UKAEA) [8]	
Penetration depth	Scabbing limit thickness
$G=3.8\times 10^{-5}\frac{NM}{D\sqrt{f_{c}}}{\left(\frac{V_{0}}{D}\right)}^{1.8}$	$\frac{s}{d}=5.3G^{0.33}$
$\frac{x}{d}=0.275-{\left[0.0756-G\right]}^{0.5}\;\left(G\leq 0.0726\right)$ $\frac{x}{d}={\left[4G-0.242\right]}^{0.5}\;\left(0.0726\leq G\leq 1.0605\right)$ $\frac{x}{d}=G+0.9395\;\left(G\geq 1.0605\right)$	$G=0.55\frac{x}{d}-{\left(\frac{x}{d}\right)}^{2}\;\left(\frac{x}{d}<0.22\right)$ $G={\left(\frac{x}{2d}\right)}^{2}+0.0605\;\left(0.22\leq \frac{x}{d}<2.0\right)$ $G=\frac{x}{d}-0.9395\;\left(\frac{x}{d}\geq 2.0\right)$

*G*: G-function, *x*: penetration depth (m), *D*, *d*: projectile diameter (m), *M*: projectile mass (kg), *V*, *V*_0_: projectile impact velocity (m/s), *f_c_*: compressive strength (Pa), *f_t_*: tensile strength (Pa), *s*: scabbing limit thickness (m), *S*: dynamic increased factor, *I*: impact factor, *N*: nose shape factor (Flat: 0.72, Blunt: 0.84, Spherical: 1.0, Sharp nose: 1.14), *N_h_*: nose shape factor of Hughes formula (Flat: 1.0, Blunt: 1.12, Spherical: 1.26, Sharp nose: 1.39).

**Table 2:** Experimental design.

Impact Condition			Specimen Dimensions		
Projectile Nose Shape	Projectile Diameter (mm)	Projectile Weight (g)	Velocity (m/s)	Size (mm)	Thickness (mm)
Sharp	25	66.8 (including carrier)	170	700 × 600 (W × H)	50, 60
Hemispherical					
Flat					

**Table 3:** Details of concrete mixes.

ID	F_ck_ (MPa)	W/B	S/a	Quantity of Materials (kg/m^3^)					Fiber	
				W	C	FA	S	G	V_f_ (%)	(kg)
NC	50	0.4	0.55	220	440	110	774	655	-	0
HSFRC									1.0	78

NC: nonreinforced concrete, HSFRC: hooked steel fiber-reinforced concrete; F_ck_: design compressive strength of concrete; W/B: water to binder ratio; S/a: the ratio of the fine aggregate volume to the total aggregate volume; W: water, C: cement, FA: fly ash, S: fine aggregate, G: coarse aggregate; V_f_: volume fraction.

**Table 4:** Mechanical properties of materials.

Materials	Mechanical Properties
Cement	Ordinary Portland cement, Density: 3.15 g/cm^3^, Fineness: 3200 cm^2^/g
Fly ash	Density: 2.20 g/cm^3^, Fineness: 3000 cm^2^/g
River sand	Density: 2.61 g/cm^3^, Water absorption: 0.81%
Gravel	Crushed gravel, Maximum size: 20 mm, Density: 2.65 g/cm^3^, Water absorption: 0.76%
Superplasticizer	Polycarboxylic acid type
Hooked steel fiber	Length: 30 mm, Diameter: 0.5 mm, Aspect ratio: 60, Density: 7.80 g/cm^3^, Tensile strength: 1140 MPa

**Table 5:** Compressive, flexural, split tensile strength of concrete at the age of 28 days.

ID	Compressive Strength (MPa)	Flexural Strength (MPa)	Split Tensile Strength (MPa)
NC	55.69	6.42	5.62
HSFRC	64.91	12.32	6.62

**Table 6:** Impact fracture on rear face.

Projectile Nose Shape	Thickness 50 mm		Thickness 60 mm	
	NC	HSFRC	NC	HSFRC
Sharp	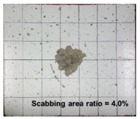	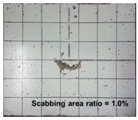	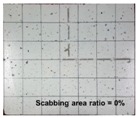	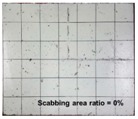
Hemi spherical	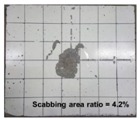	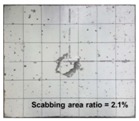	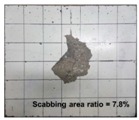	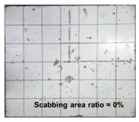
Flat